# Serum superoxide dismutase (SOD), malondialdehyde (MDA), IL-6, IL-8, TNF-a, serum immune markers after allogeneic blood transfusion in patients with gastrointestinal bleeding

**DOI:** 10.5937/jomb0-56287

**Published:** 2026-02-27

**Authors:** Lingling Wu, Zaikai Lin, Jintu Chen, Yilan Sheng

**Affiliations:** 1 Fujian Medical University Affiliated Quanzhou First Hospital Chengdong Branch, Department of Hematology, Quanzhou, China; 2 Jing'An District Centre Hospital of Shanghai (Huashan Hospital Fudan University Jing'An Branch), Department of General Surgery, Shanghai, China; 3 Quanzhou First Hospital Affiliated to Fujian Medical University, Department of Clinical Laboratory, Quanzhou, China

**Keywords:** serum superoxide dismutase (SOD), malondialdehyde (MDA), IL-6, IL-8, TNF-a, allogeneic blood transfusion, gastrointestinal bleeding, inflammatory factors, platelets function, immune globulin, serumska superoksid dismutaza (SOD), malondialdehid (MDA), IL-6, IL-8, TNF-a, alogena transfuzija krvi, gastrointestinalno krvarenje, inflamatorni faktori, funkcij'a trombocita, imunoglobulin

## Abstract

**Background:**

To analyse the changes in serum superoxide dismutase (SOD), malondialdehyde (MDA), IL-6, IL-8, TNF-a, and serum immune markers in patients with gastrointestinal bleeding (GIB) after allogeneic blood transfusion (ABT) during surgery.

**Methods:**

80 patients with GIB requiring surgical treatment were enrolled. The experimental group (EG, n=40) received 2-4 U of red blood cells during surgery, while the control group (CG, n=40) did not. T cell subsets, natural killer cells (N KC), immunoglobulin, serum inflammatory factors, platelet count, oxidative stress indicators, and hemostasis time were measured before and 1 day after ABT.

**Results:**

In the EG, NKC, CD 3+, CD 4+, and CD 4+/CD8+ decreased, IgG decreased, IL-6, IL-8, and TNF-a increased 1 day after ABT, while IgA, IgM, and platelet count remained unchanged. The EG showed a tremendous increase in SOD, a more significant decrease in MDA, and shorter hemostasis time and time for occult blood to become negative compared to the CG.

**Conclusions:**

ABT was effective in treating GIB but had an inhibitory effect on immune function. ABT did not affect platelet function but significantly changed oxidative stress indicators, inhibiting the peroxide response in GIB patients.

## Abstract (sr)

**Uvod:**

Cilj je bio da se analiziraju promene u nivou serumskih superoksid dismutaza (SOD), malondialdehida (MDA), IL-6, IL-8, TNF-a i serumskih imunoloških markera kod pacijenata sa gastrointestinalnim krvarenjem (GIB) nakon alogene transfuzije krvi (ABT) tokom operacij'e.

**Metode:**

U istraživanje je uključeno 80 pacijenata sa GIB kojima je bila neophodna hirurška intervencija. Eksperimentalna grupa (EG, n=40) primila j'e 2-4 jedinice eritrocita tokom operacije, dok kontrolna grupa (CG, n=40) nije. Pre i jedan dan nakon ABT određeni su podskupovi T limfocita, prirodne celij'e ubice (NKC), imunoglobulini, serumski inflamatorni faktori, broj' trombocita, indikatori oksidativnog stresa i vreme hemostaze.

**Rezultati:**

Kod nakon ABT, zabeleženo je smanjenje NKC, CD 3+, CD4+ i CD4+/CD8+, kao i IgG, dok su IL-6, IL-8 i TNF-a porasli. Nivoi IgA, IgM i broj' trombocita ostali su nepromenjeni. EG je pokazala veći porast SOD, veće smanjenje MDA, kraće vreme hemostaze i kraće vreme potrebno da test na okultno krvarenje postane negativan u poređenju sa CG.

**Zaključak:**

ABT je bila efikasna u lečenju GIB, ali je imala inhibitorni efekat na imunsku funkcij'u. ABT nije uticala na funkcij'u trombocita, ali j'e značaj'no promenila indikatore oksidativnog stresa, inhibirajuci peroksidativni odgovor kod pacijenata sa GIB.

## Introduction

Gastrointestinal bleeding (GIB) is a serious condition with potentially life-threatening consequences, encompassing bleeding that occurs in the digestive tract from the oesophagus to the anus. It is a leading cause of mortality [1], with a death rate of approximately 7% upon hospital admission, which can increase to 27% if bleeding occurs during hospitalisation. The primary clinical manifestation of GIB is bleeding symptoms, such as hematemesis and melena [2], often accompanied by secondary blood loss, leading to symptoms like pale complexion, fatigue, arrhythmia, low blood pressure, and even syncope. If left untreated, GIB can result in hemorrhagic shock, posing a severe threat to the patient's life and health [3][4].

GIB can be categorised into upper and lower GIB based on the bleeding site, with the Treitz ligament serving as the boundary [5]. Upper GIB, comprising about 60-70% of GIB cases, includes bleeding in the oesophagus, stomach, duodenum, pancreatic duct, bile duct, and anastomosis after gastrojejunostomy. The most common causes of upper GIB are gastric and duodenal ulcer bleeding, followed by oesophagal varices and acute gastric mucosal lesions [6][7]. Lower GIB, accounting for 20-30% of GIB cases, involves bleeding in the small intestine, colon, rectum, and other parts, with common causes including intestinal diverticulum, tumours, inflammatory bowel disease, polyps, and postoperative bleeding [8][9]. Secondary refractory ulcers in older people have become more common due to population ageing and increased use of anticoagulants, antiplatelets, and non-steroidal anti-inflammatory medicines [10].

Acute upper GIB, a typical emergency in clinical gastroenterology, is characterised by rapid onset, development, and a high mortality rate. Patients often present with varying degrees of anaemia, which can lead to hemorrhagic shock and organ failure in severe cases. Allogeneic blood transfusion (ABT) is a crucial intervention for the rescue and treatment of acute upper GIB, particularly in patients with peripheral circulatory failure, significant blood loss, and signs of hemorrhagic shock. Allogeneic blood transfusion (ABT) is one of the essential measures for rescue and treatment. When the patient suffers from acute upper GIB, if he has peripheral blood circulation failure within a short time after admission, the bleeding volume is more than 1000 mL; if the blood pressure value drops more than 15-20 mmHg when the body position changes, and there is syncope, the heart rate value increases more than 10 times per minute, the patients are considered to be suggested for acute ABT The correction of anaemia is an important method to treat such patients. At this time, ABT is necessary and plays an essential life-saving role in emergency treatment [11][12].

While essential for correcting hypovolemia and anaemia, ABT can impact the patient's immune function, leading to serum inflammation and an increased risk of infection [13][14]. This study aimed to investigate the effects of ABT on immune function, serum inflammation, platelet function, and oxidative stress indicators in patients with GIB. Specifically, we focused on the changes in serum superoxide dismutase (SOD), malondialdehyde (MDA), IL-6, IL-8, and TNF-α, which are crucial markers of oxidative stress and inflammation. SOD, an antioxidant enzyme, and MDA, a marker of lipid peroxidation, are interconnected as SOD converts superoxide radicals to hydrogen peroxide, which is then further metabolised to prevent the formation of harmful MDA. IL-6, IL-8, and TNF-α are pro-inflammatory cytokines involved in the immune response and inflammation, with IL-6 playing a role in promoting hematopoiesis and enhancing immunity. At the same time, TNF-α stimulates the expression of adhesion molecules and chemokines, leading to leukocyte accumulation at the site of inflammation.

The novelty of this study lies in its comprehensive evaluation of the effects of ABT on various immune and inflammatory markers, including SOD, MDA, IL-6, IL-8, and TNF-α, in patients with GIB. This research contributes to a better understanding of the impact of ABT on GIB patients and may help optimise blood transfusion strategies to minimise adverse effects and improve patient outcomes.

## Materials and methods

### Study design and participants

This retrospective study included 80 patients with GIB requiring surgical treatment who were admitted to the hospital between March 2018 and August 2020.

Inclusion criteria:

- Patients diagnosed with GIB based on the diagnostic criteria outlined in »Internal Medicine« and confirmed by endoscopy.
- Patients without underlying diseases such as cardiovascular, cerebrovascular, diabetes, respiratory, and endocrine diseases.
- Patients who could cooperate with all the relevant examinations in this study.

Exclusion criteria:

- Patients with congenital malformations of the digestive tract.
- Patients with bleeding caused by a lack of platelets and clotting factors.
- Patients with bleeding caused by trauma such as intubation.

### Grouping

The 80 patients were divided into two groups:

- Experimental group (EG, n=40): Received 2-4 U of red blood cells intravenously during surgery.
- Control group (CG, n=40): Did not receive allogeneic blood transfusion during surgery.

There were no significant differences in the baseline characteristics of the patients between the two groups. The study protocol was approved by the medical ethics committee of the Fujian Medical University Affiliated Quanzhou First Hospital Chengdong Branch, and informed consent was obtained from all patients and their families.

### Blood sampling and measurements

Cubital venous blood samples (4-5 mL) were collected from all patients in the morning before ABT and one day after ABT. The blood was centrifuged for 15 minutes to separate the serum. The following parameters were measured:

- T cell subsets and natural killer cells (NKC): Measured using flow cytometry.
- Immunoglobulins (IgG, IgA, IgM): Measured using immunoturbidimetry.
- Serum inflammatory factors (IL-1, IL-6, IL-8, TNF-α): Measured using enzyme-linked immunosorbent assay (ELISA).
- Platelet count: Measured using standard laboratory methods.
- Oxidative stress indicators (SOD, MDA): SOD was measured using spectrophotometric analysis, and MDA was measured using colourimetry. The detection kits were purchased from Nanjing Jiancheng Institute of Bioengineering.
- Hemostasis time: Defined as the time taken for the disappearance of coffee-like substances or blood in the excrement.
- Time for occult blood to become negative: Measured using standard occult blood tests.

The location and time of blood draw were consistent between the two groups. Table 1 Table 2

**Table 1:** The basic information of the subj'ects.

Number<br>of patients	Age <br>(years old)	Haemoglobin<br>amount at<br>admission	Weight
80 cases	34~65<br>years old	65~75 g/L	43~72 kg

**Table 2:** Experimental detection indicators and measurement methods.

Detection indicators	Experimental equipment
T cell subsets	Flow cytometer
Natural killer cells (NKC)	Flow cytometer
IL-1	Enzyme-linked <br>immunosorbent assay (ELIA)
IL-6	ELIA
IL-8	ELIA
Tumor necrosis factor -α<br>(TNF-α)	ELIA
Immune globulin	Immunoturbidimetry
Serum superoxide<br>dismutase (SOD)	Spectrophotometric analysis
Malondialdehyde (MDA)	Colourimetry

### Efficacy judgment criteria

The efficacy of the treatment was evaluated based on the following criteria:

- Markedly effective: GIB stopped without secondary bleeding within two days of treatment, and the occult blood test was negative.
- Effective: GIB stopped within three days of treatment without secondary bleeding, and the occult blood test was negative.
- Ineffective: Bleeding persisted after three days of treatment, and the occult blood test remained positive.

Total effective rate = (number of markedly effective cases + number of effective cases) / total number of cases × 100%.

### Statistical analysis

All data were analysed using SPSS 19.0 software. Measurement data were presented as mean ± standard deviation (SD), and count data were analysed using the chi-square test. A paired t-test and analysis of variance (ANOVA) were used to compare the groups. P<0.05 was considered statistically significant.

## Results

### Changes in immune cells and immunoglobulins

**Figure 1:**
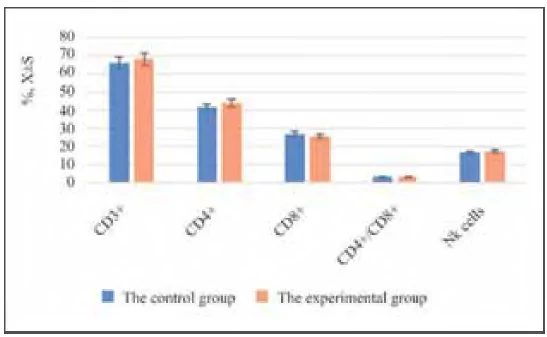
The changes of CD3^+^, CD4^+^, CD8^+^, CD4^+^/CD8^+^, and NKC prior to ABT

**Figure 2:**
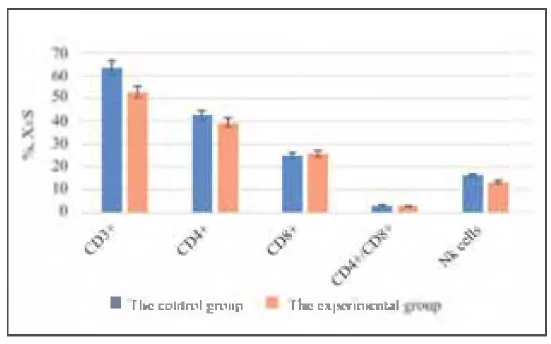
The changes of CD3^+^, CD4^+^, CD8^+^, CD4^+^/CD8^+^, and NKC 1 day after ABT

One day after ABT, the experimental group (EG) showed a significant decrease in natural killer cells (NKC), CD3^+^, CD4^+^, and CD4^+^/CD8^+^ compared to their levels before ABT (P<0.05) (Figure 1 and Figure 2).

Figure 1 and Figure 2 illustrated that in the EG 1 day after ABT, NKC, CD3^+^, CD4^+^, CD8^+^, and CD4^+^ / CD8^+^ were markedly lower than those before ABT (*P*<0.05), and there was no visible change in the CG (*P*>0.05).

**Figure 3:**
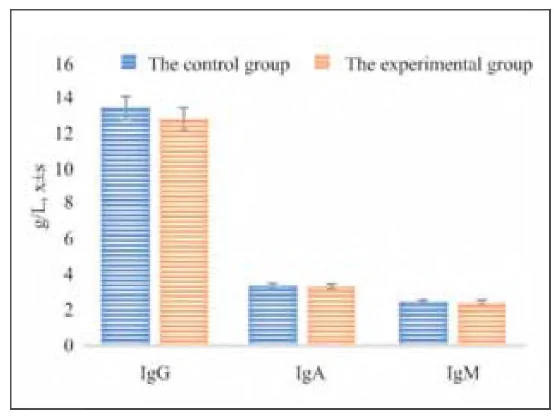
The changes of IgG, IgA, and IgM before ABT.

**Figure 4:**
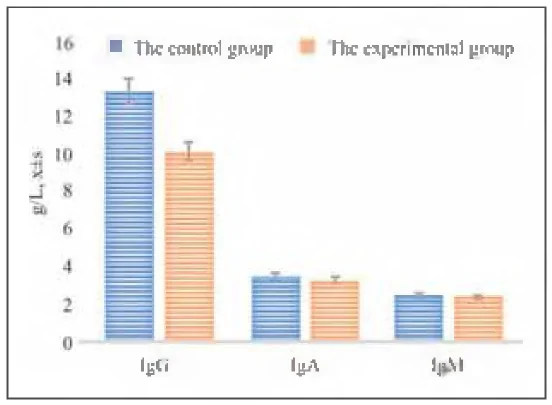
The changes of IgG, IgA, and IgM one day after ABT.

In contrast, the control group (CG) exhibited no significant changes in these parameters. The immunoglobulin G (IgG) levels were also significantly lower in the EG after ABT (P<0.05), while IgA and IgM remained relatively stable (P>0.05) (Figure 3 and Figure 4).

### Changes in serum inflammatory factors

**Figure 5:**
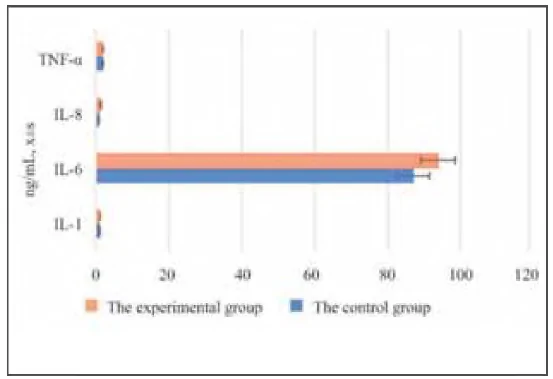
The changes of IL-1, IL-6, IL-8, and TNF-α prior to ABT

**Figure 6:**
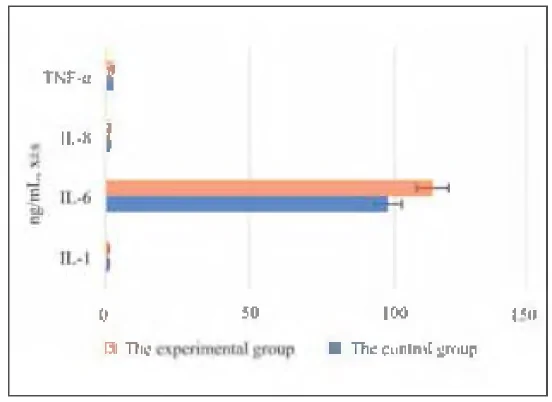
changes of IL-1, IL-6, IL-8, and TNF-α after ABT

The EG experienced a substantial increase in serum inflammatory factors, specifically IL-6, IL-8, and TNF-α, one day after ABT (P<0.05) (Figure 5 and Figure 6). However, there was no significant change in IL-1 levels before and after ABT (P>0.05).

### Platelet count

**Figure 7:**
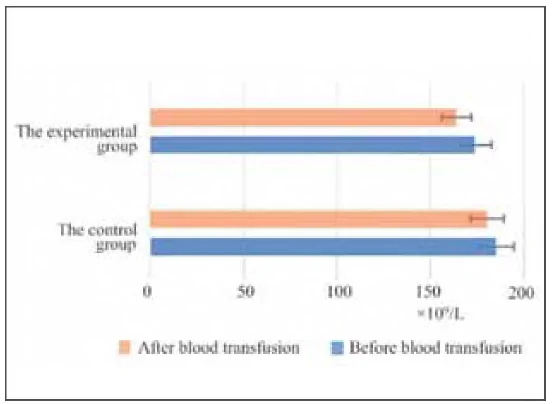
The changes in counts of platelets before ABT and one day after ABT.

The platelet count decreased in both groups before and after ABT, but the changes were not statistically significant (P>0.05) (Figure 7).

### Oxidative stress indicators

**Figure 8:**
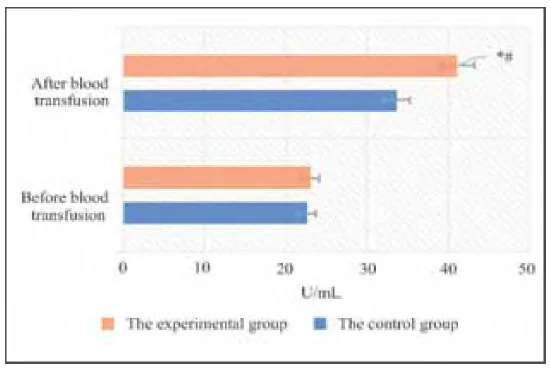
The changes in SOD and MDA before ABT. Note: *# suggested the distinction was statistically obvious (*P*<0.05).

**Figure 9:**
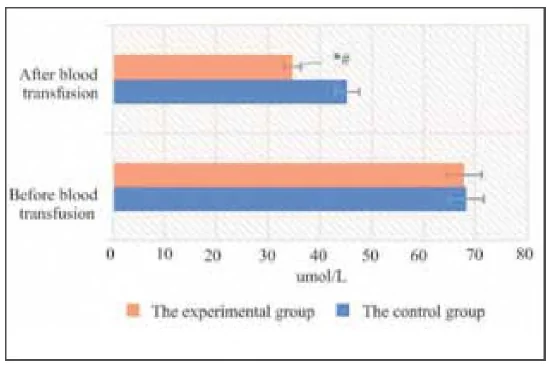
The changes in SOD and MDA after ABT. Note: *# suggested the distinction was statistically obvious (*P*<0.05).

Before ABT, there was no significant difference in SOD and MDA levels between the EG and CG (P>0.05). However, after ABT, the EG showed a greater increase in SOD and a more significant decrease in MDA compared to the CG (P<0.05) (Figure 8 and Figure 9).

### Hemostasis time and time for occult blood to become negative

**Figure 10:**
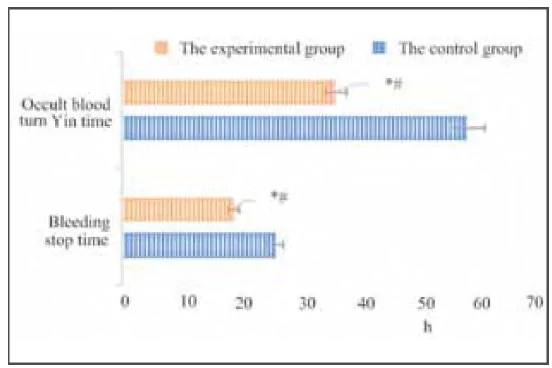
The changes in hemostasis time of the subjects. Note: *# suggested the distinction was statistically obvious (*P*<0.05)

The EG exhibited a shorter hemostasis time and time for occult blood to become negative compared to the CG (P<0.05) (Figure 10).

These results suggest that while effective in treating GIB, ABT impacts immune function, inflammation, and oxidative stress. Further discussion will elaborate on the implications of these findings.

## Discussion

GIB is a common disease of the digestive system in clinics, and there are many reasons for bleeding. Therefore, it has a very high morbidity. Acute massive bleeding can lead to hemorrhagic shock. In this emergency, allogeneic blood transfusion treatment should be performed in addition to surgical hemostasis treatment [15]. In recent years, due to the short-age of blood sources, the increased awareness of diseases caused by ABT, and the in-depth study of ABT's effects on the body's immune function. Some scholars have clarified higher requirements for blood transfusion indications in clinical operations. Leukocytes in blood components mainly mediate the immune regulation induced by ABT, and many scholars have confirmed this as immunosuppression [16]. Related literature reports that in the process of ABT, if the patient is directly infused with red blood cells or blood products containing red blood cells, it is possible to produce allogeneic antibodies against exogenous red blood cell antigens in the patient's immune system, resulting in ABT adverse events, and even worse, which will endanger the life of patients. Boateng et al. (2019) [17] believed that even if ABT can be performed preventively in advance after matching red blood cell antigens, red blood cell allogeneic immunity will inevitably occur; it showed that a large number of foreign antigens (soluble and cell-related) are introduced into the body of the same ABT, and the antigens that continue to exist in the circulation may lead to the down-regulation of the body's immune function. That was, the main manifestations are immunosuppression, inability to respond, and clone elimination. Recent studies further highlight that oxidative stress markers like SOD and MDA, as well as pro-inflammatory cytokines (IL-6, IL-8, TNF-α), are critical indicators of ABT-related immune dysregulation [18].

T lymphocytes play an indispensable role in body immunity. Related literature mentions that CD3^+^ T cells represent the overall level of cellular immunity, and CD4^+^ T cells belong to effector T cells. Its main task is assisting other cells in the immune response. Cytotoxic T cells are mainly CD8^+^ T cells, whose function is primarily to inhibit other immune cells. The critical indicator that reflects the body's immune status is the ratio between CD4^+^ / CD8^+^, and the change in the ratio can directly reflect the immune function status of the body's cells [19][20]. NKCs are natural immune cells that significantly influence the body's immune process. NKC can react quickly to virus-infected cells and mutated cancer cells. This response is mainly done by killing and secreting cytokines [21]. In the experiment, on the first day after ABT, NKC, CD3^+^, CD4^+^, and CD4^+^/ CD8^+^ were markedly reduced in the EG compared with those before aBt (*P*<0.05), while the CG had no visible change. These findings align with studies demonstrating ABT-induced suppression of NKC activity and T-cell subsets in surgical cohorts [22].

Immune globulin plays an essential role in humoral immunity. Some researchers pointed out that the IgG content in serum is high and widely distributed. It is the primary immune globulin produced during the humoral immune response when the body is infected with the pathogen for the second time. It has the functions of anti-infection, neutralising endotoxin and regulating the body. Its decrease indicates that the patient's anti-infection ability has decreased. IgA in serum has anti-bacterial, virus, endotoxin and other effects, and its decrease suggests that the patient's anti-infective and anti-viral abilities have decreased simultaneously [23][24]. In the experiment of this article, the IgG of the EG decreased after 1 day of ABT, which may be caused by the ageing of the red blood cells stored in the blood during the storage process, the effects of various antigens produced by the blood cells, and the reduction of CR1. Several bodily disorders' onset and progression are also obviously influenced by serum inflammatory factors. Literature has confirmed that IL-6, the most common clinical cytokine, promotes hematopoiesis and enhances immunity. It primarily plays a biological role inducing T cell activation and B cells to produce antibodies. At the same time, IL-6 also participates in the body's inflammatory response process [25].

On the other hand, TNF-α can stimulate vascular endothelial cells to express adhesion molecules, making it easy to adhere to leukocytes, thereby simulating the monocyte-macrophages to secrete chemokines so that white blood cells accumulate at the site of inflammation. These three cytokines are important inflammatory factors that initiate the anti-bacterial inflammatory response. Increasing the activity of TNF-α can help inhibit and kill bacteria, improve the patient's resistance to infection and speed up the ability of wound healing [26][27]. However, IL-8 levels remained stable in our cohort, diverging from reports of IL-8 elevation in trauma patients receiving ABT, which may reflect differences in patient populations or transfusion protocols.

Platelets have a significant role in the process of blood coagulation played by the body. In recent years, it has been reported that the membrane phospholipid surface of platelets provides the coagulation reaction site, and the activated platelets and the microparticles produced by lysis have the protective effect of coagulation and hemostasis. When the counts of platelets are more significant than 100 X 10^9^/L, it indicates that the body has no bleeding tendency. When the count of platelets is about 65 X 10^9^/L or less than 65 X 10^9^/L, it suggests that the body has a bleeding tendency. When a patient receives a large amount of red blood cell suspension, it can reduce the dilution of platelets and coagulation factors, resulting in blood oozing on the wound surface [28][29]. In this article, the counts of platelets of the two groups of patients before ABT and one day after ABT decreased, but the index changes were not obvious (*P*>0.05), which may be related to the number of red blood cells infused by the patient and the preservation time of the infused red blood cells. SOD is the key enzyme to remove oxygen free radicals in the body, while MDA is the product of oxygen free radicals, both of which are essential indicators reflecting the body's oxidative stress state [14][15]. Our findings of elevated SOD and reduced MDA post-ABT (*P*<0.05) align with studies linking ABT to transient oxidative stress mitigation, though prolonged RBC storage may reverse this effect [30]. In this article, the degree of SOD increase and MDA decrease of the EG after receiving ABT during the surgery were higher as against the CG (*P*<0.05). Such results suggest that intraoperative ABT can markedly inhibit the peroxide reaction of patients with upper GIB.

Therefore, for ABT during surgery, the clinical indications for blood transfusion and the amount of blood transfusion should be strictly controlled. Once the condition improves, other methods can be used to correct the anaemia, thereby reducing the suppression of the patient's immune function through intraoperative ABT, reducing the patient's economic burden and alleviating the shortage of blood resources. In summary, intraoperative ABT will stimulate the patient to produce a certain inflammatory response, which has a particular inhibitory effect on the patient's immune function. However, intraoperative ABT within a certain range has no visible impact on the patient's platelet count and function. On the contrary, after intraoperative ABT, the patient's oxidative stress indicators will change markedly; that is, it can inhibit the peroxide response of patients with upper GIB.

## Dodatak

### Conflict of interest statement

All the authors declare that they have no conflict of interest in this work.
